# Assessment and maintenance of normal fluid status in older people living in care homes: a scoping review protocol

**DOI:** 10.1136/bmjopen-2024-095874

**Published:** 2026-02-27

**Authors:** Catherine Fielding, Sevim Yasemin Hodge, Eleanor Lunt, Gerri Mortimore, Benjamin Edward Smith, Sarah Jane Ashworth, Rachael Carroll, Clare Sobieraj, Claire McGuire, Alice Bloxham, Emma Cooper James, Ruqayya Hamza, Leah Torr, Heather Buchanan, Helen Hurst, Adam L Gordon

**Affiliations:** 1 Research and Innovation, Healthcare of Older People, Nottingham University Hospitals NHS Trust, Nottingham, UK; 2 Centre for Rehabilitation and Ageing Research, University of Nottingham Faculty of Medicine and Health Sciences, Nottingham, UK; 3 Wolfson Institute of Population Health, Queen Mary University of London, London, UK; 4 Academic Centre for Healthy Ageing, Barts Health NHS Trust, London, UK; 5 Nottingham University Hospitals NHS Trust, Nottingham, UK; 6 Division of Rehabilitation and Ageing, University of Nottingham School of Medicine, Nottingham, UK; 7 University of Derby, Derby, UK; 8 Physiotherapy Outpatients, Derby Teaching Hospitals NHS Foundation Trust, Derby, UK; 9 Exemplar Healthcare, Sheffield, UK; 10 Carer Representative, Lincolnshire, UK; 11 Acute Kidney Injury Nurse, University Hospitals of Derby and Burton NHS Foundation Trust, Derby, UK; 12 Department of Medicine for the Elderly, University Hospitals of Derby and Burton NHS Foundation Trust, Derby, UK; 13 University Hospitals of Derby and Burton NHS Foundation Trust, Derby, UK; 14 University of Nottingham, Nottingham, UK; 15 School of Health and Society, University of Salford, Salford, UK; 16 Renal, Salford Royal Hospital, Northern Care Alliance NHS Foundation Trust, Salford, UK

**Keywords:** Aged, Frail Elderly, GERIATRIC MEDICINE, Nursing Homes

## Abstract

**Abstract:**

**Introduction:**

Maintaining normal fluid status is critical to health and well-being. Older people are vulnerable to abnormal fluid status and associated complications, morbidity and mortality. Care home residents are especially vulnerable due to dependence on care home staff, frailty and multiple comorbidities. However, it is unclear what evidence-based assessments and interventions are available to support effective fluid-status management in care homes.

**Methods and analysis:**

This article describes a protocol for a scoping review aiming to explore current evidence on assessment and maintenance of normal fluid status in older people living in care homes. It is written in line with Preferred Reporting Items for Systematic Reviews and Meta-Analyses-Scoping Reviews guidelines. The literature search will cover 10 clinical databases and include research registries and grey literature, and reference lists of included articles. Screening of sources will comprise two stages: title and abstract and full-text screening. Articles will be managed in Covidence and data charting will use bespoke data extraction forms used to generate a narrative synthesis of results. Screening and data extraction will be completed by two coauthors, blinded to each other’s results, with discrepancies adjudicated by a third author when required.

**Ethics and dissemination:**

As a scoping review of existing evidence, rather than conducting new research, ethical approval is not required. Dissemination will comprise a peer-reviewed publication, presentation at a national conference focused on care of older people, and local patient and public involvement and engagement groups. We will explore opportunities to develop care home-facing education materials working with our local Applied Research Collaboration.

**Registration:**

Open Science Framework: https://doi.org/10.17605/OSF.IO/AVS5B.

**STRENGTHS AND LIMITATIONS OF THIS STUDY**

- The application of scoping review methodology to the design of this protocol aims to ensure the review produces results that are relevant and applicable to guide future research and practice.
- The breadth of coauthors, including experts in clinical and academia and engaging stakeholders, has strengthened the design of this scoping review.
- The absence of critical appraisal in the planned scoping review meets the aims and objectives of the scoping review, ensuring no relevant articles are excluded, but means the scoping review will provide no guide as to the robustness of sources.
- The scoping review is restricted to English language articles for pragmatic reasons, which may mean relevant articles published in another language are excluded.

## Introduction

 Maintaining normal fluid status within the body is a basic but critical function to maintain health. A healthy body balances the competing activities of fluid intake and fluid excretion to achieve a normal fluid status (euvolaemia). The thirst mechanism triggers the oral intake of fluids,^1 2^ while the kidneys excrete excess fluid, using processes within the nephron to ensure the required amount of fluid is excreted.^2^ These mechanisms are regulated through multiple hormones including the renin–angiotensin–aldosterone system (RAAS)^3 4^ and anti-diuretic hormone (ADH) (aka vasopressin).^5^ These hormonal systems are complex, altering electrolyte and fluid retention and excretion in the nephrons, using vasoconstriction to increase blood flow and blood pressure and driving thirst.^3^,^5^ While these processes aim to regulate fluid status in the body, fluid is also lost from the body in an unregulated manner through insensible losses, mainly via the gut, lungs and skin.^2^ Maintaining normal fluid status within the body is complex and easy to decompensate and become abnormal. As people age, the ability to maintain fluid homeostasis deteriorates as a consequence of multiple physiological changes. Thirst responses are blunted in older people, ADH levels are altered and the ability of kidneys to clear creatinine and concentrate urine decreases with age.^6^

Dehydration is a lack of total body water, which may be caused by: poor fluid intake; increased insensible fluid losses due to, for example, excess sweating, vomiting or diarrhoea; of a combination of both poor fluid intake and increased insensible fluid losses.^7^ Alongside fluid imbalance, people with dehydration may fail to consume, or lose excessive amounts of plasma electrolytes, the most important of which is sodium. Depending on relative depletion of sodium and body water, the patient may present in a hypertonic or isotonic state—with slightly different indications for treatment. Regardless, dehydration causes multiple problems. It increases the risk of mortality,^1 7 8^ although much evidence is based on observational data. Dehydration is also associated with significant morbidity. The ‘National Confidential Enquiry into Patient Outcomes and Deaths’ (NCEPOD) report into acute kidney injury (AKI) found that dehydration was the most common cause of AKI,^9^ causing hypoperfusion of the nephrons from reduced vascular volume. Delirium can also develop following dehydration,^1 7^ although one may intensify the other, where delirium may also reduce oral intake and thus exacerbate or cause dehydration. Dehydration is also associated with other complications, especially in older people, including: increasing the risk of both arterial and venous thrombosis causing strokes, acute coronary syndrome, deep vein thrombosis and pulmonary embolisms; heart failure; infection especially urinary tract infections; orthostatic hypotension; falls and constipation.^1 7 10^ While dehydration can be corrected with intravenous fluids and electrolytes, for older people, it is often viewed as a significant problem that is preventable by encouraging adequate oral fluid intake, with or without electrolytes, depending on the clinical scenario.^1 9 10^

Fluid overload is another form of fluid abnormality, where excess fluid in the body is not excreted efficiently. This can result in fluid being deposited in the tissues (peripheral oedema), lungs (pulmonary oedema or pleural effusions) or abdomen (ascites).^4^ While fluid overload can be associated with overadministration of fluids,^2^ the kidneys are normally able to excrete excess fluid efficiently, unless administered to excess or if pathological processes interfere with the excretion of fluid. However, if the kidneys are not working correctly (ie, chronic kidney disease, AKI), then they may not excrete excess fluid efficiently. Fluid overload is often associated with heart failure, which causes decreased perfusion of the kidneys but may also interfere with the RAAS and ADH systems, further preventing the excretion of excess fluid.^4^ To add complexity to fluid excretion, fluid within the body is also distributed in three compartments—intravascular, interstitial and intracellular.^7^ As fluid is removed from the vascular space by the kidneys, oncotic pressure then pulls fluid from the other spaces to ‘refill’ the vascular space and remove excess fluid from these spaces.^11^ However, if this oncotic pressure is compromised due to sepsis or low plasma protein levels (mainly albumin), then interstitial and intracellular fluid does not move to the vascular space through this mechanism and extracellular fluid is harder to remove^4^. Therefore, fluid overload is not just about whether the kidneys are working but also whether the fluid can get to the nephrons, via the vascular system, to be removed. As fluid overload is often caused by abnormal pathology within the body, it is harder to prevent. While treating reversible causes (eg, low albumin, AKI) can resolve fluid overload, many causes are not so easily reversible. Treatment through diuretics is often the main focus, occasionally alongside other medications, to promote diuresis.^4^

### Abnormal fluid status in older people

Maintaining normal fluid status in older people is challenging. Factors that increase their vulnerability to dehydration include:

- A blunted thirst response, meaning the physiological prompt to drink reduces, reducing their oral intake.^2 7 12^
- The fear of incontinence and urinary frequency, alongside decreased mobility and increased dependency to get to the toilet, discouraging older people from drinking.^1 7 10 13^
- Reduced cognition leading to forgetting when they last had a drink and lack of recognition that they are thirsty, losing further cues to drink.^1 10 14^
- Dependency on others to provide drinks due to both reduced cognition and mobility.^1^
- Multimorbidity and polypharmacy, especially diuretics, which are often not reviewed as people become increasingly frail, altering their need for these medications.^2 7 13^
- Dysphagia.^1^

While dehydration is of concern in older people, the risk of fluid overload is also higher due to a higher prevalence of heart failure^4^ and physiological changes that predispose them to fluid retention.^2^ There are multiple factors that can contribute to abnormal fluid status in older people, alongside physiology that is less adaptable to compensate for this. Therefore, the vulnerability of older people to abnormal fluid status is evident.

Further to this, older people who live in care homes appear to have further vulnerability to abnormal fluid status, especially dehydration. Their need for care home support likely means they are increasingly frail and more prone to the vulnerabilities that all older people experience. The reliance on care home staff to provide drinks means they do not always get drinks when they need them and their individual preferences for drinks are not always recognised, further discouraging oral fluid intake.^9^ The incidence of dehydration in care home residents was higher than older people living in their own homes,^13^ estimated as up to 38%.^14^ Therefore, older people who live in care homes are particularly vulnerable to abnormal fluid status.

Interventions are needed to recognise and prevent abnormal fluid status in older people, particularly those living in care homes. Very simply, preventing or correcting fluid abnormality early in the person’s home helps prevent escalation, preventing associated complications and hospital admissions. Care home staff are ideally placed to manage this in residents’ day-to-day lives. However, assessment of fluid status is important to enable care home staff to recognise and promptly correct potential abnormal fluid status. There is a current debate around what parameters should be used to do this.^7^ A Cochrane review of 24 papers looking at the diagnostic utility of clinical signs and tests found that individual symptoms, signs and tests had little of no diagnostic use and highlighted combinations of tests for future evaluation.^15^ While international guidelines highlight the further challenges presented by dementia, which is prevalent in long-term care settings.^16^ Interventions need to focus on those that care home staff can action promptly and as part of the resident’s normal life. Paulis *et al*^17^ completed a Delphi survey to consensus on pragmatic diagnosis of dehydration in care home residents in the care home context. However, this only defines a strategy, rather than addressing the validation or implementation of this strategy. Educational packages aimed at care home staff are another approach, assuming that educating care home staff will remove some of the barriers to hydration in older people.^10^ Other strategies to address barriers may include regular toileting to reduce concern about continence and urinary frequency, regular offer of drinks, offering preferred drinks and offering food that contained fluid (eg, soup).^1 10^ A descriptive literature has aimed to describe how older people consume drinks in care homes,^18^ while number of initiatives have sought to develop and implement structured approaches to fluid intake in care home residents. These have included training interventions^19^ and quality improvement studies,^20^ which have yielded some insights about how to support fluid intake in care homes. A small cluster-randomised feasibility RCT of a multicomponent intervention to support hydration found the intervention to be feasible and acceptable but no follow-up definitive trial has yet been conducted.^21^ A recent realist evaluation focused primarily on preventing urinary tract infections in the care home setting, considered evidence from 56 published papers and highlighted two relevant context–mechanism–outcome configurations as part of wider programme theory^22^—one relating to prioritising hydration in care homes, the other to systems that drive action helping residents to drink more.

Managing fluid overload in care homes is a slightly different proposition from preventing dehydration. Approaches to prevent dehydration have included, for example, whole home strategies to increase the availability and variety of fluids available, coupled to behavioural change strategies to promote staff to offer fluid more often.^20^ This often comes with caveat that individual care planning is needed but with the emphasis, in the published literature at least, on individualising approaches of how staff get people to drink.^21^ Structured approaches to avoid fluid overload in susceptible people may involve, by contrast, exempting residents from such regimens and focusing behavioural strategies on regulating and/or actively discouraging fluid intake.

The corollary of this published literature is that, while targets for intervention have been developed, no definitive approach to fluid management in the care home setting has been agreed or published.

### Rationale

Despite the importance of maintaining normal fluid status in older people living in care homes, well-evidenced interventions to achieve this are lacking and current reviews on this subject are sparse. A recent systematic review by Parkinson *et al* found the prevalence of low intake dehydration among care home residents to be 34%.^23^ A separate systematic review by Paulis *et al* identified numerous diverse risk factors associated with dehydration, with a lack of consistency between studies.^14^ However, their conclusions do not provide any guidance on how to maintain normal fluid status, only providing detail on the scope of the problem. Hodgkinson *et al*^13^ completed a systematic review exploring the maintenance of oral hydration in older adults, identifying that research evidence in this area was poor. Since this review in 2003, the management of hydration has changed and there is likely more research available, particularly considering the release of the NCEPOD report in 2009.^9^

It continues to be unclear what evidenced assessments and interventions exist to maintain and correct fluid status in older people living in care homes, with many recommendations based on expert opinion rather than evidence. Therefore, a scoping review is needed to enable understanding of all the research in this field. Scoping reviews provide a comprehensive review of a subject, whereas systematic reviews tend to be more focused on a specific area or intervention.^14 24^ A scoping review exploring all relevant evidence into maintaining normal fluid status in older people living in care homes will enable understanding of pre-existing assessments and interventions and identify where gaps exist in the evidence.

## Methods and analysis

The scoping review protocol was developed using Preferred Reporting Items for Systematic Reviews and Meta-Analyses extension for scoping reviews^25^ and the Joanna Briggs Institute guidance.^24^ The completed protocol was registered on the Open Science Framework platform and is accessible at https://doi.org/10.17605/OSF.IO/AVS5B. Throughout the description of this protocol, terminology coherent with scoping reviews has been used for consistency. This has been divided into the sections required in a scoping review.

### Patient and public involvement

Carer and care home staff representatives have been involved in developing the research question and eligibility criteria, ensuring this is meaningful for older people living in care homes. They have often shared the challenges of maintaining a normal fluid status for older people who are becoming increasingly frail. This has helped to form the rationale for this review. Some of these representatives are keen to participate in the review and have been named as coauthors. For carers, we have agreed their main focus of contribution will be in identifying and interpreting key results, which will be obtained through discussion with the lead author. To facilitate involvement of these representatives, extra information will be provided as needed alongside bespoke discussions with the lead author.

### Research question, aims and objectives

The following research question guides the scoping review:

‘How can normal fluid status be assessed and maintained in older people living in a care homes?’

This scoping review aims to explore current evidence base on the assessment and maintenance of normal fluid status, including prevention of fluid abnormality, in older people living in care homes. To support this question and provide further focus, five objectives are summarised in table 1.

**Table 1:** Objectives for the scoping review

Objective 1	To summarise current research and identify gaps in research to underpin assessment and maintenance of normal fluid status, including prevention of fluid abnormality, in older people living in care homes.
Objective 2	To identify assessment techniques of normal fluid status, including prevention of abnormal fluid status, in older people living in care homes.
Objective 3	To identify interventions aimed at maintaining normal fluid status, including preventing fluid abnormality, in older people living in care homes.
Objective 4	To understand experiences of older people living in care homes who undergo assessment or interventions to maintain normal fluid status, prevent fluid abnormality or experience fluid abnormality.
Objective 5	To identify outcomes measured in sources that explore assessment and maintenance of normal fluid status, including prevention of fluid abnormality, in older people living in care homes.

#### Eligibility criteria

Eligibility criteria provide further definition and clarity to the scoping review question, providing focus in the literature search and screening process. The Participants, Concept and Context, as recommended for scoping reviews,^24 25^ provides further context for eligibility criteria. The type of evidence sources^24^ and limits placed on the literature search are defined within eligibility criteria.

##### Participants

This scoping review focuses on sources that include people 65 years and older. This will include sources that solely meet this criterion but also those sources with a broader population where there is a subgroup analysis that is performed in those 65 years or older. This is expected to identify sources relevant to the scoping review question. However, if there is a shortage of sources, consideration will be given whether to expand this criterion to include sources with an average population age of 65 years and older.

##### Concept

This scoping review focuses on sources that examine the assessment and maintenance of normal fluid status, in part or in full, including prevention of abnormal fluid status. Normal fluid status was defined to include maintenance of euvolaemia, where a normal fluid status is maintained, although is not exclusive to euvolaemia and may also include normal hydration. Relevant sources may not solely focus on the maintenance of normal fluid status but also on correcting abnormal fluid status, which includes dehydration and/or fluid overload. Again, the focus was on sources where participants had an abnormal fluid status, not a specific definition of dehydration or fluid overload.

Sources will focus on assessments and interventions. Assessment may include monitoring of input, clinical signs of abnormal fluid states (eg, blood pressure, dry mucous membranes) and diagnostic tests (eg, point of care tests). It is also recognised that sources may discuss nutrition in conjunction with hydration; therefore, sources that focus on hydration as part of nutrition will be included. However, sources solely focusing on nutrition will be excluded.

##### Context

This scoping review includes sources set in the care home setting, which includes both residential and nursing homes within the UK and the equivalent in other international sources. For the purposes of this scoping review, a care home is defined as a long-term residence of people requiring supervision and assistance with day-to-day living to the extent they live in a group building rather than a separate building (eg, warden controlled flat). This will include respite care, where residents stay overnight, but exclude day care where they do not stay overnight. Some care homes may provide day care as well as residents who stay overnight, which will not be excluded, but those sources in settings that solely provide day care will be excluded. Care home can include terms like nursing home, long-term care, care home, residential home, residential facility, institutional care, skilled nursing facility, institutionalisation, care facility, homes for the aged and continuing care,^26^ and many more terms. This scoping review will exclude sources that do not exclusively focus on the care home setting, including sources about people who live in care homes and are admitted to hospital. If there is a shortage of sources, those that include both people who live in care homes and those who do not, or sources that focus on people who live in care homes but are in hospital, may be considered for inclusion.

##### Types of evidence sources

The types of sources used in this scoping review are summarised in box 1.

Box 1 **Types of evidence sources to be included in the review**

All types of research including primary and secondary research and quantitative and qualitative research designs.

Published case studies.

Published quality improvement and service evaluation.

PhD thesis identified through clinical database searches.

Published clinical practice guidelines and recommendations identified through clinical database searches.

Protocols of reviews, research, service evaluation and quality improvement project registered on clinical database searches.

This scoping review will exclude conference abstracts and government reports. It will exclude reviews that do not include systematic or scoping review methodology. It is recognised that qualitative studies may be exploratory, so if they contain findings related to residents’ experiences of assessment or interventions to maintain normal fluid status, prevent fluid abnormality or experience fluid abnormality, then these studies will be included even if this was not the aim of the study.

##### Limits

This review will limit the search to studies completed or published in the last 15 years. Healthcare’s approach to hydration has also changed in response to the NCEPOD report to prevent AKI, released in the UK in 2009.^9^ While this was a UK initiative, other initiatives in other countries also happened at similar times. This changed the landscape of fluid management. This limit of the last 15 years will ensure that the review only includes sources relevant to the current time. This review will also only include studies available in English. This is a pragmatic limit due to having no access to translation services.

### Literature search

The literature search strategy was developed with support of a clinical librarian.

#### Information sources

The clinical databases to be included in the literature search are outlined in table 2. This included both medical databases and also those with articles relevant to nursing and allied health professionals. To ensure a comprehensive search strategy, a list of research and trial registries were also identified to be included in the literature search, which is also included in table 2. Reference lists from any review identified through these searches that incorporates a systematic literature search strategy will be searched. Articles identified through this method will be added to the screening articles.

**Table 2:** Information sources to be used in the literature search

Clinical Databases	Cumulative Index of Nursing and Allied Health Literature (CINAHL) (1937–present)
	EMCARE (1995–present)
	PsychInfo (1806–present)
	Medline (1946–present)
	Pubmed National Centre for Biotechnology Innovation/National Library of Medicine (1946–present)
	EMBASE (1974–present)
	Cochrane Database of Systematic Review (CDSR) (1996–present)
	Allied and Complementary Medicines Database (AMEDP (1985–present)
	Social Policy and Practice (SPP) (1890s–present)
	HMIC (Health Management Information Consortium) (1979–present)
Research and Trials Registries	Cochrane Central Register of Controlled Trials (CENTRAL)
	NIHR Be Part of Research (UK Trials)
	ISRCTN Registry
	PROSPERO
	ICRTP (International Clinical Trials Registry Platform)
	Clinicaltrial.gov
	Research Registry
	Open Source Framework
	Figshare
	Joanna Briggs Institute (JBI) Evidence Based Practice Database

#### Search strategy

The clinical database search was developed using various terms identified from the eligibility criteria (table 3). Once the search strategy was developed, this was piloted on one database (MedLine—box 2) and the first 100 references were screened for relevance. This indicated that the search strategy identified relevant studies, but also helped to clarify eligibility criteria. For databases and registries without an advanced search strategy (eg, research registries), a less detailed search using a selection of these terms from the clinical database search strategy (box 2) will be used to refine.

**Table 3:** Terms used to develop the clinical database search strategy

Population	Old* Elder* Frail* Ageing / Aging Aged Geriatri* Centarian* Nonagenarian* Octogenerian* Senior*
Concept	Euvolaem* Euvolem* Normovoleam* Normovolem* Hydrat* Euhydrat* Dehydrat* Hypovolaem* Hypovolem* Fluid overload Oedema Oedema Fluid retention Fluid retain* Fluid defici* Fluid Intoxificat* Hypervolaem* Hypervolem* Effusion Water stress
Context	Social care Care home Nursing home Long-term care Residential home Residential facility Skilled nursing facility Institution* Care facility Homes for the aged Continuing care Intermediate care Extended care

Box 2 **Database search piloted in Medline**

1. exp Aged/
2. (older* or aged or ag?ing or elder* or frail* or geriatri* or centenarian* or nonagenarian* or octogenarian* or senior*).ti,ab,kw.
3. or/1–2
4. exp Residential Facilities/
5. ((residential* or care* or nursing* or group* or age* or geriatri* or extended care* or intermediate care*) adj3 (home* or facilit*)).ti,ab,kw
6. senior housing.ti,ab,kw
7. (social care* or continuing care* or long term care* or long-term care*).ti,ab,kw
8. or/4–7
9. 3 and 8
10. Organism Hydration Status/
11. (euvolem* or euvolaem*).ti,ab,kw
12. (normovolem* or normovolaem*).ti,ab,kw.
13. hydrat*.ti,ab,kw.
14. euhydrat*.ti,ab,kw.
15. ((fluid* or water*) adj3 (content* or status* or level*)).ti,ab,kw.
16. Dehydration/
17. dehydrat*.ti,ab,kw.
18. water stress*.ti,ab,kw.
19. Hypovolemia/
20. (hypovolem* or hypovolaem*).ti,ab,kw.
21. (hypervolem* or hypervolaem*).ti,ab,kw.
22. Water Intoxication/
23. EdemaOedema/
24. (edemaoedema or oedema).ti,ab,kw.
25. effusion.ti,ab,kw.
26. ((fluid* or water*) adj3 (overload* or retain* or retention* or intoxicat* or defici*)).ti,ab,kw.
27. or/10–26
28. 9 and 27
29. limit 28 to english language
30. limit 29 to last 15 years

#### Screening

Screening will be completed in two consecutive stages:

1. Title and abstract screening.
2. Full-text screening.

At each stage of screening, two coauthors will screen articles. During screening, each coauthor will be blinded to the other’s screening results until they have completed their own. Results will then be reviewed and disagreement between coauthors will be discussed. If the two coauthors cannot reach an agreement, then this will be reviewed by a third coauthor who will act as adjudicator and make the final decision about inclusion. During title and abstract screening, if it is unclear whether the study meets eligibility criteria, it will be included in full-text screening. A list of questions were developed to assist during screening and ensure clarity between coauthors (box 3). These questions will be refined if required, during the screening process, to ensure screening identifies sources relevant to the eligibility criteria. Currently, the same questions are planned for each stage of the screening process, but it is recognised that titles or abstracts may not provide clarity on all these questions. These studies will then progress to full-text screening. If the study meets the criteria in part, then it will be included but, if possible, only the results relevant to the eligibility criteria will be included in the final results.

Box 3 **Screening questions**

1. Is the source published primary or secondary research, published quality improvement, published service evaluation, published clinical practice guideline/recommendation, a case study or a protocol of these?
  - If no, exclude.
2. Does the source population include only people over 65 years old?
  - If no, is there a subgroup analysis of people over 65 years old?
  - If no, exclude.
3. Does the source focus solely on people on a fluid restriction, including those on dialysis?
  - If yes, exclude.
4. Does the source focus solely on people with lymphoedema?
  - If yes, exclude.
5. Does the source aim, fully or in part, to assess fluid status, maintain normal fluid status, prevent abnormal fluid status or understand residents’ experiences of assessment or maintenance of fluid status?
  - If no, exclude.
6. Does the source solely focus on an intervention that requires a medical prescription to administer (eg, medication, intravenous fluids)?
  - If yes, exclude.
7. Does the source only include people who live in a care home or respite settings?
  - If no, is there a subgroup analysis that solely includes people from care home or respite settings?
  - If no, exclude.

Critical appraisal of sources is not planned, as no sources will be excluded based on quality. While critical appraisal can be part of a scoping review, this is only recommended if required to meet specific objectives of the review.^25 26^ In this review, this is not required and may risk excluding important sources. Therefore, critical appraisal is excluded to ensure a comprehensive review of the current literature and enable understanding of what is currently known and easily identify gaps in knowledge.

### Data charting

A bespoke form will be created to record and standardise data charting extracted from the sources included in the scoping review. Different bespoke forms will be used for different types of sources. The planned content of each bespoke form is outlined in table 4. While bespoke forms will be used to guide and standardise data charting, all forms will include a free-text section for coauthors to add any further relevant points. As the type of sources that will be included in the scoping review is not known at this stage, each bespoke form will be developed and reviewed by at least two coauthors once the screening of sources has been completed. Each form will be piloted on two relevant sources, before widespread use within the scoping review. Data charting will be performed by two coauthors independently and blinded to each other’s results until it is completed. Any discrepancies in data charting will be discussed between the two coauthors initially. If the two coauthors cannot reach an agreement, then this will be reviewed by a third coauthor who will act as adjudicator and make the final decision as to the correct data charting.

**Table 4:** Proposed contest of data extraction forms

Primary research and case studies	The population within the study The aims of the study The methods used Description of any interventions used The verbatim results related to fluid status, including quantitative results and descriptions of themes and subthemes from qualitative research Conclusions drawn and any recommendations for practice
Secondary research	The aim of the review Methods used to complete the review The results of the review Any conclusions and recommendations for practice
Published QI and service evaluation	The aim of the project Methods used to complete the project The results of the project Any conclusions and recommendations for practice
Clinical practice guidelines and recommendations	The overall aim of the guideline Methods for development of the guideline Any guideline/recommendation statements relevant to the scoping review Any rationale attached to relevant guideline/recommendation statements
Protocol	The aim of the study The methods of the study The plan for implementation including any link to included publications or sources that provide the results of the study

### Synthesis of results

The results of the literature search and screening will be reported, as recommended by the PRISMA extension for scoping reviews.^25^ The results of database searches, numbers of included and excluded articles and reasons for exclusions will be summarised in a PRISMA flowchart. The data charted from included sources will be summarised in a narrative synthesis. This will include the characteristics of included studies and their findings. This narrative synthesis will likely be split into sections for sources that examine the different objectives of the scoping review and the different types of sources. The results of sources will be described but will not be synthesised together by meta-analysis or meta-synthesis. All data from sources that are relevant to the scoping review aims and objectives will be included in any report of findings, within the limitations of word restrictions, but irrelevant results to the aims and objectives will be excluded.

## Ethics and dissemination

As this is a scoping review of projects previously completed by others and available for review, either through publication or grey literature, ethical approval is not required. However, to ensure this research is of good quality and generates findings relevant and true to both the previous research conducted and clinical practice, the author group has attempted to adhere scoping review methodology and best practice to design the protocol. Implementation of the scoping review will adhere to the protocol, as closely as possible and deviations from the protocol will be recorded for transparency.

Following completion of the scoping review, this is planned for publication in a peer-reviewed journal. Results will also be disseminated through national conferences and local research and patient and public involvement and engagement groups. This scoping review is designed to identify gaps in current understanding for future and as such will also inform the design of a future research project.
