# Supplementary material for: Assessment and maintenance of normal fluid status in older people living in care homes: a scoping review protocol
Source: BMJ Open. 2026 Feb 27;16(2):e095874. doi: 10.1136/bmjopen-2024-095874 (PMC12958877; doi:10.1136/bmjopen-2024-095874)
Supplement: Supplementary file 1 [file bmjopen-2024-095874.reviewer_comments.pdf]

# PEER REVIEW HISTORY

BMJ Open publishes all reviews undertaken for accepted manuscripts. Reviewers are asked to complete a checklist review form and are provided with free text boxes to elaborate on their assessment. These free text comments are reproduced below.

## ARTICLE DETAILS

### Title (Provisional)

Assessment and Maintenance of Normal Fluid Status in Older People Living in Care Homes: A Scoping Review Protocol

### Authors

Fielding, Catherine; Hodge, Sevim; Lunt, Eleanor; Mortimore, Gerri; Smith, Benjamin Edward; Ashworth, Sarah Jane; Carroll, Rachael; Sobieraj, Clare; McGuire, Claire; Bloxham, Alice; Cooper James, Emma; Hamza, Ruqayya; Torr, Leah; Buchanan, Heather; Hurst, Helen; Gordon, Adam L

---

## VERSION 1 - REVIEW

---

| | |
|---|---|
| **Reviewer** | **1** |
| **Name** | **Bunn, Diane** |
| **Affiliation** | **University of East Anglia, Norwich Medical School** |
| **Date** | **02-Dec-2024** |
| **COI** | **None** |

---

Thank you for asking me to review this protocol.
I am particularly interested in this area of work, and the authors are addressing a major issue in older adults. However, I thought that the introduction needed greater clarification about the two main types of dehydration (and they also mentioned fluid overload) and that the review of the literature should have more breadth and depth, by including a review of more current works and primary evidence (many of the references were old). In particular around care home interventions and methods of assessing dehydration (both types). As such, the 'gap in knowledge' and the rationale for this review was not really very clear: 'What do we know, what don't we know, what is unclear, and what would we like to know? I think the introduction needs more focus and clarity, to provide the evidence and rationale for the research questions. I also thought that this Scoping Review was very broad in its remit (even for a scoping review!) and perhaps it may be more doable if it was more focused.

I suppose I also struggled with the term 'deranged' which is used throughout, and wonder if it is possible to find an alternative term? Also, replace 'the elderly' with 'older adults'..

| Page | Line | Section | Comment |
|---|---|---|---|
| | | Abstract | I would suggest that he abstract needs to include some further key information:<br>(i) mention the underpinning methodology<br>(ii) the writing of the protocol is inline with PRISMA ScR guidelines.<br>(iii) There is a sub-title of 'dissemination' but nothing about dissemination is mentioned!<br>(iv) data management, eg covidence or similar |

| | | | |
|---|---|---|---|
| 3 | 32 | Introduction | • lines 61-63: I think it should be clearer that these mechanisms deteriorate in older adults:, ie thirst mechanism is ineffective in older adults, renal concentrating ability reduces, reduction of ADH and osmoreceptor responses[1–5]<br>• lines 73-77. There is now good evidence that we should discriminate between low-intake dehydration (hypertonic – not drinking enough, low fluid, electrolytes stable) and hypovolaemia (isotonic, low fluid AND electrolytes, due to D&V, sweat, haemorrhaging etc) so perhaps discuss in more detail here as to different types, causes and management?[6–9]<br>• Lines 77-91. The evidence for increased risk of comorbidities relating to dehydration is more nuanced, I would say, and relates very much to the type of dehydration (low-intake or hypovolaemia) so perhaps clarify this in the arguments being made. Most of the evidence comes from observational studies.<br>• Line 146: the most recent prevalence data is by Parkinson et al [10]<br>• There have been a number of research projects in care homes, so perhaps include a wider discussion of these, including a discussion of the interventions developed, in the introduction? I am thinking particularly of the groups at West London (Wilson, Bak), Northumbria (Cook, Hodges) & UEA (Bunn, Hooper).<br>• There have been a number of more recent reviews than the one undertaken by Hodgkinson.<br>• Given the research Q is around recognising and assessing normal fluid status in older care home residents, the introduction should |
| | | | include a discussion on the evidence to date on the robustness of current tools to assess fluid intake. See for example [4–7,11,12]<br>• Overall, I think the scope of this scoping review is very wide by looking at both low-intake and hypovolaemia types of dehydration, as well as fluid overload. As such, I think it would be helpful if the distinctions and the arguments around causes, risks, management could be clearer between the two throughout the introduction. For examples the problems associated with overhydration are very different to those with low-intake hydration. |

| | | | |
|---|---|---|---|
| 5 | | Methods | • Whilst I thought the research questions were clear, the underpinning rationales and arguments for exploring these further were not really covered in the introduction.<br>• If this is about older adults in care homes, then this should be mentioned in the eligibility criteria (just mentions older adults).<br>• It is not clear why residents undergoing fluid restriction will not be eligible, when the introduction talked about those on diuretics etc, which are for CCF presumably? And the ScR topic is 'fluid derangement'? I think the rationale around this should be clarified.<br>• Similarly re 'prescribed diagnostic tests', it is not clear why these would be excluded, only based on that they are not doable in care homes. Given that current evidence would indicate that only directly-measured serum osmolality or calculated serum osmolarity (using Krajuria Krahn equation) are the only robust tests of dehydration in older adults, do you not need to include these papers too, and actually exclude the others? What would be your rationale for including any other tests? [6,7,9,12,13]<br>• Whilst I understand that formal critical appraisal is not a feature of ScR's perhaps some mention about approaching the literature critically, rather than descriptively would be good? |

***References:***


1. Cheuvront SN, Kenefick RW, Charkoudian N, Sawka MN. Physiologic basis for understanding quantitative dehydration. Am J Clin Nutr. 2013;97:455–62.

2. Hooper L, Bunn DK, Abdelhamid A, Gillings R, Jennings A, Maas K, et al. Water-loss (intracellular) dehydration assessed using urinary tests : how well do they work? Diagnostic accuracy in older people. Am J Clin Nutr. 2016;104:121–31.

3. Hooper L, Bunn DK, Downing A, Jimoh F, Groves J, Free C, et al. Which frail older people are dehydrated? The UK DRIE study. J Gerontol A Biol Sci Med Sci. 2016;71(10):1341–7.

4. Hooper L, Abdelhamid A, Attreed NJ, Campbell WW, Channell AM, Chasagne P, et al. Clinical symptoms, signs and tests for identification of impending and current water-loss dehydration in older people. Cochrane Database Sytematic Rev [Internet]. 2015;(4):Art. No.: CD009647. Available from: http://onlinelibrary.wiley.com/doi/10.1002/14651858.CD009647

5. Bunn DK, Hooper L. Signs and Symptoms of Low-Intake Dehydration Do Not Work in Older Care Home Residents - DRIE Diagnostic Accuracy Study. J Am Med Dir Assoc [Internet]. 2019; Available from: https://doi.org/10.1016/j.jamda.2019.01.122

6. Lacey J, Corbett J, Forni L, Hooper L, Hughes F, Moss C, et al. A multidisciplinary consensus on dehydration: definitions , diagnostic methods and clinical implications. Ann Med [Internet]. 2019;51(3–4):232–51. Available from: https://doi.org/10.1080/07853890.2019.1628352

7. Volkert D, Beck AM, Faxén-Irving G, Frühwald T, Hooper L, Keller H, et al. ESPEN guideline on nutrition and hydration in dementia – Update 2024. Clin Nutr. 2024;43(6):1599–626.

8. Frith J. New horizons in the diagnosis and management of dehydration. Age Ageing. 2023;52:1–6.

9. Beck AM, Seemer J, Knudsen AW, Munk T. Narrative review of low-intake dehydration in older adults. Nutrients. 2021;13(9):1–16.

10. Parkinson E, Hooper L, Fynn J, Wilsher SH, Oladosu T, Poland F, et al. Low-intake dehydration prevalence in non-hospitalised older adults: Systematic review and meta-analysis. Clin Nutr [Internet]. 2023;42(8):1510–
20. Available from: https://doi.org/10.1016/j.clnu.2023.06.010

11. Fortes MB, Owen J a., Raymond-Barker P, Bishop C, Elghenzai S, Oliver SJ, et al. Is This Elderly Patient Dehydrated? Diagnostic Accuracy of Hydration Assessment Using Physical Signs, Urine, and Saliva Markers. J Am Med Dir Assoc [Internet]. 2015;16:221–8. Available from: http://linkinghub.elsevier.com/retrieve/pii/S1525861014006148

12. Volkert D, Beck AM, Cederholm T, Cruz-Jentoft A, Goisser S, Hooper L, et al. ESPEN guideline on clinical nutrition and hydration in geriatrics. Clin Nutr [Internet]. 2018; Available from: https://doi.org/10.1016/j.clnu.2018.05.024

13. Siervo M, Bunn DK, Prado C, Hooper L. Accuracy of prediction equations for serum osmolarity in frail older people with and without diabetes. Am J Clin Nutr. 2014;100:867–76.

---

## VERSION 1 - AUTHOR RESPONSE

| Reviewer 1 comments: | | |
|---|---|---|
| Comment raised i | Response by author i | Location of revisions i |
| Abstract:<br><br>(i)<br>mention the underpinning methodology<br><br>(ii)<br>the writing of the protocol is inline with PRISMA ScR guidelines.<br><br>(iii)<br>There is a sub-title of 'dissemination' but nothing about dissemination is mentioned!<br><br>(iv)<br>data management, eg covidence or similar | Thankyou.<br><br>(i)<br>We are not clear what the reviewer means by underpinning methodology. The methodology here is Scoping review, which is clearly stated and by allusion to the PRISMA-ScR guidelines, we've made it clear what the methodological parameters are.<br><br>(ii)<br>We have now specifically included reference to PRISMA ScR in the abstract.<br><br>(iii)<br>We have added the following about dissemination: "Dissemination will comprise a peer-reviewed publication, presentation at a national conference focussed on care of older people, and local patient and public involvement and engagement groups. We will explore opportunities to develop care home facing education materials working with our local Applied Research Collaboration."<br><br>(iv)<br>We have stated that we will manage articles in Covidence. | Abstract, page 1 |

| | | |
|---|---|---|
| I suppose I also struggled with the term 'deranged' which is used throughout, and wonder if it is possible to find an alternative term? Also, replace 'the elderly' with 'older adults'.. | We have replaced the term "deranged" with "abnormal" throughout, and "elderly" with "older people". | Throughout article. |
| Introduction:<br>(i)<br>lines 61-63: I think it should be clearer that these mechanisms deteriorate in older adults:, ie thirst mechanism is ineffective in older adults, renal concentrating ability reduces, reduction of ADH and osmoreceptor responses[1–5]<br><br>(ii)<br>lines 73-77. There is now good evidence that we should discriminate between low-intake dehydration (hypertonic – not drinking enough, low fluid, electrolytes stable) and hypovolaemia (isotonic, low fluid AND electrolytes, due to D&V, sweat, haemorrhaging etc) so perhaps discuss in more detail here as to different types, causes and management?[6–9]<br><br>(iii)<br>Lines 77-91. The evidence for increased risk of comorbidities relating to dehydration is more nuanced, I would say, and relates very much to the type of dehydration (low-intake or hypovolaemia) so perhaps clarify this in the arguments being made. Most of the evidence comes from observational studies.<br><br>(iv)<br>Line 146: the most recent prevalence data is by Parkinson et al [10] | (i)<br>Thank you – we have added the following: "As people age, the ability to maintain fluid homeostasis deteriorates as a consequence of multiple physiological changes. Thirst responses are blunted in older people, ADH levels are altered, and the ability of kidneys to clear creatinine and concentrate urine decreases with age."<br><br>(ii)<br>Thank you for the opportunity to capture more nuance here. Arguably fluid and electrolyte status is more complex than the peer-reviewer alludes to and the binary approach they suggest is an oversimplification. We have aimed to capture this nuance as follows:<br>"Dehydration is a lack of total body water which may be caused by: poor fluid intake; increased insensible fluid losses due to for example excess sweating, vomiting or diarrhoea; of a combination of both poor fluid intake and increased insensible fluid losses. Alongside fluid imbalance, people with dehydration may fail to consume, or lose excessive amounts of plasma electrolytes, the most important of which is sodium. Depending on relative depletion of sodium and body water, the patient may present in a hypertonic or isotonic state – with slightly different indications for treatment"<br><br>(iii)<br>We disagree with the peer reviewer – the association is pretty robust regardless of the type of dehydration considered. We have added the phrase: "Regardless of cause"<br><br>(iv)<br>We thank the peer reviewer for directing us to their more recent publication. We have added this as follows:<br>"A recent systematic review by Parkinson et al, found the prevalence of low intake | (i)<br>Introduction paragraph 1<br><br>(ii)<br>Introduction paragraph 2<br><br>(iii)<br>Introduction Paragraph 2<br><br>(iv)<br>Rationale Paragraph 1 |

| | | |
|---|---|---|
| | dehydration amongst care home residents to be 34%” | |
| (v)<br>There have been a number of research projects in care homes, so perhaps include a wider discussion of these, including a discussion of the interventions developed, in the introduction? I am thinking particularly of the groups at West London (Wilson, Bak), Northumbria (Cook, Hodges) & UEA (Bunn, Hooper). | (v)<br>Thankyou, we have included the following in the text:<br>“A descriptive literature has aimed to describe how older people consume drinks in care homes, whilst number of initiatives have sought to develop and implement structured approaches to fluid intake in care home residents. These have included training interventions and quality improvement studies, which have yielded some insights about how to support fluid intake in care homes, A small cluster randomised feasibility RCT of a multicomponent intervention to support hydration found the intervention to be feasible and acceptable but no follow-up definitive trial has yet been conducted. A recent realist evaluation focussed primarily on preventing Urinary Tract Infections in the care home setting, considered evidence from 56 published papers and highlighted two relevant context-mechanism-outcome configurations as part of wider programme theory – one relating to prioritising hydration in care homes, the other to systems that drive action helping residents to drink more.”<br><br>Then two paragraphs later:<br><br>“The corollary of this published literature is that, whilst targets for intervention have been developed, no definitive approach to fluid management in the care home setting has been agreed or published.” | (v)<br>Abnormal fluid status in older people, para 5 and 7 |
| (vi)<br>There have been a number of more recent reviews than the one undertaken by Hodgkinson. | (vi)<br>Thankyou – we have deleted the reference to this paper and have cited more recent literature, as highlghted elsewhere in our responses to the reviewer. | |
| (vi)<br>Given the research Q is around recognising and assessing normal fluid status in older care home residents, the introduction should a discussion on the evidence to date on the robustness of current tools to assess fluid intake. See for example [4–7,11,12] | (vi)<br>Thank you, we have included the following: “A Cochrane review of 24 papers looking at the diagnostic utility of clinical signs and tests found that individual symptoms, signs and tests had little of no diagnostic use, and highlighted combinations of tests for future | (vi)<br>Abnormal fluid status in older people, para 4 |

| | | |
|---|---|---|
| (vii)<br>Overall, I think the scope of this scoping review is very wide by looking at both low-intake and hypovolaemia types of dehydration, as well as fluid overload. As such, I think it would be helpful if the distinctions and the arguments around causes, risks, management could be clearer between the two throughout the introduction. For examples the problems associated with overhydration are very different to those with low-intake hydration. | evaluation. Whilst international guidelines highlight the further challenges presented by dementia, which is prevalent in long-term care settings"<br><br>(vii)<br>Thank you. We have added the following:<br><br>"Managing fluid overload in care homes is a slightly different proposition from preventing dehydration. Approaches to prevent dehydration have included, for example, whole home strategies to increase the availability and variety of fluids available, coupled to behavioural change strategies to promote staff to offer fluid more often. This often comes with caveat that individual care planning is needed but with the emphasis, in the published literature at least, on individualising approaches of how staff get people to drink. Structured approaches to avoid fluid overload in susceptible people may involve, by contrast, exempting residents from such regimens and focussing behavioural strategies on regulating and/or actively discouraging fluid intake." | (vii)<br>Abnormal fluid status in older people, para 5 |
| Methods<br><br>(i)<br>Whilst I thought the research questions were clear, the underpinning rationales and arguments for exploring these further were not really covered in the introduction.<br><br>(ii)<br>If this is about older adults in care homes, then this should be<br>mentioned in the eligibility criteria (just mentions older adults).<br><br>(iii)<br>It is not clear why residents undergoing fluid restriction will not be eligible, when the introduction talked about those on diuretics etc, which are for CCF presumably? And the ScR topic is 'fluid derangement'? I think the rationale around this should be clarified. | (i)<br>Thankyou. We have substantively modified the introduction in line with the peer-reviewer's. We trust that the rationale is now clearer.<br><br>(ii)<br>Thankyou. We have described the full eligibility criteria using the PCC (person, concept, context pneumonic). Care homes are clearly mentioned under context – thus reading the eligibliity in criteria it should be clear that the review will include older people living in care homes.<br><br>(iii)<br>Thank you. We had initially excluded these studies as we felt that fluid restriction was used for heterogenous group of conditions ranging from psychogenic polydipsia through Syndrome of Inappropriate Antidiuretic Hormone secretion, to heart failure and renal failure. We have, though, considered this at some length and, on balance, a signification amount of the literature related to fluid overload is likely to focus | |

| | | |
|---|---|---|
| | on fluid restriction.  We have removed this exclusions criterion.  We are confident we can work through the heterogeneity at review stage. | |
| (iv)<br>Similarly re ‘prescribed diagnostic tests’, it is not clear why these would be excluded, only based on that they are not doable in care homes. Given that current evidence would indicate that only directly-measured serum osmolality or calculated serum osmolarity (using Krajuria Krahn equation) are the only robust tests of dehydration in older adults, do you not need to include these papers too, and actually exclude the others? What would be your rationale for including any other tests? [6,7,9,12,13] | (iv)<br>Thank you.  We have removed this exclusion criterion. | |
| (v)<br>Whilst I understand that formal critical appraisal is not a feature of ScR’s perhaps some mention about approaching the literature critically, rather than descriptively would be good? | (v)<br>Thank you.  Critical thinking is of course key to reading any literature, but we don’t plan to rewrite established scoping review methodology as part of this study.  We have not modified the text in response to this comment. | |
